# A comparison of the protein-coding genomes of two green sulphur bacteria, *Chlorobium tepidum* TLS and *Pelodictyon phaeoclathratiforme* BU-1

**DOI:** 10.1186/s13104-015-1535-8

**Published:** 2015-10-14

**Authors:** Kristin M. Wreggelsworth, Daniel Barker

**Affiliations:** School of Biology, University of St Andrews, St Andrews, Fife KY16 9TH UK

**Keywords:** *Chlorobium tepidum* TLS, *Pelodictyon phaeoclathratiforme* BU-1, Chlorobiaceae, Protein families, Comparative genomics, OrthoMCL, Ortholog, Paralog, Raspberry Pi

## Abstract

**Background:**

*Chlorobium tepidum* and *Pelodictyon phaeoclathratiforme* are organisms within the green sulphur bacteria family, Chlorobiaceae, occupying very different habitats. It has recently been proposed that the genera *Chlorobium* and *Pelodictyon* are synonymous.

**Results:**

To investigate generic boundaries for the two species, protein families were predicted computationally based on sequence similarity across the genome-wide protein sets of *Chlorobium tepidum* TLS and *Pelodictyon phaeoclathratiforme* BU-1. The distribution of the resulting protein families across the two species was summarized. The largest number of families exhibited 1:1 putative orthology between the two species (1468 families). Of families unique to one of the species, the largest number was unique to *P. phaeoclathratiforme* (113 families), of which the largest family contained pentapeptide repeat proteins (16 proteins). Families unique to *P. phaeoclathratiforme* also included a family of gas vesicle synthesis proteins (four proteins). Although only seven families were identified as containing paralogous proteins in both species (with two or more proteins in each species), this group included families of major biochemical importance. One such family, with three members in each species, contained magnesium chelatase, an enzyme involved in the chlorophyll biosynthetic pathway.

**Conclusion:**

The unique protein family groups in both *C. tepidum* and *P. phaeoclathratiforme* mirror the occupancy of different environments, while key shared family groups provide evidence for a common origin for the species, as previously suggested in the literature. The current study only uses sequence similarity-based protein families for the two species. This, alone, does not permit a firm conclusion to be drawn on the taxonomic question, of whether the two species belong in one genus or two.

**Electronic supplementary material:**

The online version of this article (doi:10.1186/s13104-015-1535-8) contains supplementary material, which is available to authorized users.

## Background

The family Chlorobiaceae, commonly known as green sulphur bacteria, has a complex taxonomic history. Organisms have conventionally been classified within this family by morphological and phenotypic characteristics [1]. This anoxygenic phototrophic bacterial family uniquely contains chlorosomes, structures for harvesting light. Fenna–Matthews–Olson protein, another protein unique to this family’s system, is then used to mediate the transfer of energy between the chlorosomes and photosynthetic reaction centre. More recently, researchers have looked beyond morphological and phenotypic characteristics to identify relationships within this family through phylogeny reconstructions based on the sequences of the Fenna–Matthews–Olson protein and 16S rRNA [2]. Originally, within this family there have been the genera *Chlorobium* and *Pelodictyon.* As a result of these molecular phylogenetic analyses, there has been a shift to regard these two genera as synonymous [1, 2]. To analyse this relationship on a broader genomic scale, the current study examines protein family membership across the genome-wide protein sets of one strain from each of the original genera, *Chlorobium tepidum* TLS *and Pelodictyon phaeoclathratiforme* BU-1.

Since these two bacteria are found within the same family, and arguably within the same genus, they have very similar morphological, phenotypic and genotypic characteristics. The distinctions between the two provide insight into their evolutionary history and key characteristics of the species.

*P. phaeoclathratiforme* BU-1 was first identified in 1989 as a strain of rod-shaped single celled bacteria. At the time of species specification, the presence of a gas vacuole, its immobility and its characteristic net-like colony structure identified it as a member of *Pelodictyon.* Although it is a green sulphur bacterium, it is brown in colour. It is similar in morphology, cytology and physiology to *P. clathratiforme* in all but its photosynthetic pigments, the latter displaying the green phenotype [3]. The genome contains over 3,000,000 base pairs, with approximately 2700 protein-coding genes [4].

*C. tepidum* was first isolated in 1991 in hot springs of high acidity and sulphide composition. The TLS strain was sequenced in 2002 [5]. It is the only thermophilic *Chlorobium*, optimally growing at a temperature of 48 °C. Its circular DNA contains over 2 million base pairs, with approximately 2250 protein-coding genes [5].

To investigate the extent of genome-wide similarities and differences between the two species, sequence similarity-based protein families were predicted using OrthoMCL, which delimits groups of similar proteins on the basis of BLAST results. OrthoMCL does not assign proteins present in only one copy in a single species to a family [6].

Analysis of these two species, through the protein families for which their genomes code, will provide insight into their defining characteristics, and constitutes preliminary research on the taxonomic standing of the two genera.

## Results

Investigation of the two green sulphur bacteria, *C. tepidum* TLS *and P. phaeoclathratiforme* BU-1, was done through the analysis of sequence similarity-based protein families delimited by OrthoMCL. Where a protein family spanned both species, the members in one species were assumed to be orthologs of the members in the other species. Where a protein family contained multiple proteins within one species, these proteins were assumed to be paralogs.

Under these assumptions, there were a larger number of families of paralogous proteins unique to *P. phaeoclathratiforme* (113 families) than those families that solely contain proteins from *C. tepidum* (13 families). Within these purely paralogous families, those found in *P. phaeoclathratiforme* were larger than those of *C. tepidum*, with mean protein counts per family 3.186 ± 2.32 (SD) and 2.308 ± 0.63, respectively (Table 1).

**Table 1:** Comparisons of the spread of counts of sequence similarity-based protein families across *Chlorobium tepidum* (‘cct’) and *Pelodictyon phaeoclathratiforme* (‘ppb’), for Uniprot protein sets

	Unique for cct	Unique for ppb	1 copy for ppb, multiple copies for cct	1 copy for cct, multiple copies for ppb	Multiple copies for both species	1 copy for both species	Ungrouped proteins
Within conditions							
# of families	13	113	7	33	7	1468	–
# of proteins	30	360	24	109	32	2936	1444
# of cct proteins	30	–	17	33	15	1468	687
Percentage of cct proteins (%)	1.33	–	0.756	1.47	0.667	65.2	30.5
# of ppb proteins in the condition	–	360	7	76	17	1468	757
Percentage of ppb proteins (%)	–	13.4	0.261	2.83	0.633	54.7	28.2
Within each family group							
Mean ± SD # of cct proteins	2.308 ± 0.63	–	2.429 ± 0.77	1 ± 0	2.143 ± 0.38	1 ± 0	–
Mean ± SD # of ppb proteins	–	3.186 ± 2.32	1 ± 0	2.303 ± 0.81	2.429 ± 0.79	1 ± 0	–

The paralogous families found unique to one species give insight into molecular pathways important to the survival of that particular species. The fact that the family is unique to the species suggests it may have a role in its environment, not relevant to the environment of the other species. That there are paralogs, rather than a single copy, could indicate subfunctionalisation, neofunctionalisation, or the requirement for a high level of gene expression. For example, OrthoMCL Group 1 contains 16 pentapeptide repeat proteins from the *P. phaeoclathratiforme* genome-wide protein set (Table 2; each family has an arbitrary group number, assigned by OrthoMCL and unique within this study; groups are given in Additional file 1). They are predicted to have a beta-helix structure [7], but the function of these proteins has yet to be identified. These proteins have been identified in cyanobacteria, bacteria and plants, however they are absent in *C. tepidum* [7].

**Table 2:** The main function and protein counts of sequence similarity-based protein families between *Chlorobium tepidum* (‘cct’) and *Pelodictyon phaeoclathratiforme* (‘ppb’), for Uniprot protein sets

Group^a^	Total proteins^d^	cct proteins	ppb proteins	Proposed function^b^
1	16	0	16	Pentapeptide repeat protein
3	10	0	10	Transcriptional regulator, XRE family
7	7	0	7	PAS/PAC sensor signal transduction histidine kinase
8	7	1	6	TIR domain protein, TPR repeat-containing protein, SEFIR domain protein
10	7	0	7	Transcriptional regulator, XRE family
12	6	3	3	Magnesium-protoporphyrin methyltransferases, magnesium chelatase
13	6	2	4	Multi-sensor histidine kinase
18	6	0	6	Plasmid maintenance system antidote protein, XRE family
19	5	4	1	Outer surface protein, putative, Hemagglutinin-related protein, Tia invasion determinant-related protein
20	5	0	5	Putative transcriptional regulator
21	5	1	4	Transcriptional regulator, XRE family^c^
22	5	0	5	HipA domain protein
23	5	0	5	Ribonuclease VapC (RNase VapC)
24	4	2	2	Transposase
25	4	2	2	Filamentation induced by cAMP protein Fic, Death-on-curing family protein^c^
26	4	2	2	Excinuclease ABC, A subunit
27	4	2	2	Bche/P-methylase family protein, Radical SAM domain protein
28	4	3	1	Flp/Fap pilin component^c^
31	4	2	2	ATP-dependent zinc metalloprotease FtsH
32	4	1	3	Probable pyruvoyl-dependent arginine decarboxylase
34	4	1	3	Sel1 domain protein repeat-containing protein^c^
35	4	0	4	RNA-directed DNA polymerase
37	4	0	4	YapH protein
38	4	0	4	Gas vesicle synthesis GvpLGvpF
40	4	0	4	PilT protein domain protein
41	4	0	4	Transposase IS4 family protein

^a^The group number has been arbitrarily assigned by the OrthoMCL program. Groups are provided in Additional file 1

^b^Functional annotation was taken from the UniProt database (http://www.uniprot.org) and from [4] and [5], with manual integration of information where it varied within a family. Any group that contained only uncharacterised protein functions were excluded from this table

^c^Some of the proteins within this family group have yet to be characterised

^d^Only groups containing four or more proteins were included

There is also a family of paralogous proteins unique to *P. phaeoclathratiforme* which contain vesicle synthesis proteins (Table 2). *P. phaeoclathratiforme* uses the gas vesicle as buoyancy control in the water [3]. Although the presence of a gas vesicle is no longer used to identify genera, it is a characteristic of *P. phaeochlathratiforme* that, as seen from our results as well as the literature [5, 8], is not found in *C. tepidum.*

The greatest number of families exhibited a 1:1 orthologous relationship between the species (1468 families, including 54 % of *P. phaeoclathratiforme* proteins and 65.2 % of *C. tepidum* proteins; Table 1). This large number of 1:1 orthologs supports that these two species share a common origin. This is to be expected, especially as they are now classed within the same genus [2].

There were a larger number of families containing multiple *C. tepidum* proteins and only one *P. phaeoclathratiforme* protein, than families containing multiple *P. phaeoclathratiforme* proteins and one *C. tepidum* protein (Table 1). Overall, neither of these conditions were very prevalent; 0.756 % of *C. tepidum* and 0.261 % of *P. phaeoclathratiforme* proteins were found in the groups containing multiple copies of *C. tepidum* and 1.47 % of *C. tepidum* and 2.83 % of *P. phaeoclathratiforme* proteins were found in the groups containing multiple copies of *P. phaeoclathratiforme*. An example is Group 19, which contains one *P. phaeoclathratiforme* protein and four *C. tepidum* proteins (Table 2). These are beta-barrel structural membrane proteins, some of which are hemagglutinin-related proteins [4, 5]. The increase in membrane protein paralogs in *C. tepidum* may be correlated with the structural integrity of the membrane and the ability to live as a thermophile.

There were also very few families containing paralogs for both species (7 families, 0.667 % of *C. tepidum* proteins and 0.633 % of *P. phaeoclathratiforme* proteins; Table 1). One example is Group 12, which contains magnesium chelatases and methyltransferases involved in the chlorophyll and bacteriochlorophyll biosynthetic pathways [4, 5]. Multiple paralogs in an ortholog group suggest a speciation event happened in evolutionary history after multiple gene-specific duplications took place (i.e. these sequences are out-paralogs [9]), or that the gene-specific duplications were novel to each species and resulted in subfunctionalisation or neofunctionalisation separately.

The results of this study confirm the relationship between the two green sulphur bacteria, *C. tepidum* TLS and *P. phaeoclathratiforme* BU-1, as well as highlighting defining characteristics of the two.

Our analysis is preliminary. We have used only sequence similarity-based protein families delimited by OrthoMCL to make inferences about orthology and paralogy. Other methods could lead to different results [10]. Beyond this methodological point, our suggestions for further work include analysis of the ungrouped proteins (30.5 % of *C. tepidum* proteins and 28.2 % of *P. phaeoclathratiforme* proteins; Table 1). These are not represented in the protein families analysed in the current paper, but may play a role in the overall differences between the two species. A function-specific search through the groups would also provide greater insight into the well-known differences between the two species, for example in photosynthetic pigments. Comparisons of other species within this combined genus will also lead to a greater understanding of the extent of similarities and differences in gene content; a similar analysis could be performed for two entirely different genera of bacteria within another family, to provide a base-line example of the extent of variation in gene content within and between two accepted genera. Using other proteins, unrelated to 16S rDNA and Fenna-Matthews-Olson protein, for the production of phylogenetic trees will increase the reliability of the results, as there were still some discrepancies between the phylogenetic trees produced [2]. This would then lead to a stronger basis for classification and taxonomy.

## Methods

For the delimitation and investigation of protein families, analyses were performed using the 4273*π* variant of the Raspbian Linux operating system [11] running on Raspberry Pi Model B hardware.

### Acquisition of the genome-wide protein sets

The Fasta-format protein sets of *C. tepidum* and *P. phaeoclathratiforme* were downloaded from the UniProt database (http://www.uniprot.org; Additional files 2, 3) in early October 2014. For comparison, protein sets were also downloaded from Ensembl Genomes (Release 23, http://ensemblgenomes.org; Additional files 4, 5). Results reported are based on the UniProt data, however the complementary files for analyses using Ensembl Genomes data are also provided (Additional files 4, 5, 6, 7, 8, 9, 10, 11, 12).

### Protein family delimitation

The genome-wide protein sets of the two species were analysed using OrthoMCL software (http://orthomcl.org) [6] with MCL [12]. Steps were followed as laid out in the protocol from the OrthoMCL User Guide (also available at http://orthomcl.org), using default parameters with the exception that the ‘all-versus-all’ NCBI BLAST [13] was run with the BLOSUM45 substitution matrix. The sequence similarity-based protein families (‘orthologous groups’) output by OrthoMCL were stored in a file, groups.txt (Additional file 1). The OrthoMCL output was verified by bl2seq searches on random groups (selected using the random function in Microsoft Excel), on the expectation that sequences within a group should show strong evidence of homology. This was the case (*E* = 10^−119^, 8 × 10^−78^, and 6 × 10^−63^ and identity = 85, 48 and 33 % for Groups 119, 696 and 96 respectively).

### Protein family analysis

Since OrthoMCL only produces families of proteins, Perl scripts were written [14] to analyse their distribution across the two species (Additional files 13, 14, 15, 16, 17, 18). Counts were verified using a script written independently [15]. Protein functions and structures were obtained through Web access to the Uniprot (http://www.uniprot.org) and InterPro (http://www.ebi.ac.uk/interpro) databases in November 2014.

### Protein function analysis

The function of any protein family containing four or more proteins within a group was analysed. Any protein group that contained exclusively uncharacterised proteins, of which there are 16 groups, was excluded from the results in Table 2 and assigned to Table S1 (Additional file 19).

## Additional files

10.1186/s13104-015-1535-8 groups.txt. Predicted protein families (groups) output by OrthoMCL for *C. tepidum* and *P. phaeoclathratiforme* UniProt protein sets.
10.1186/s13104-015-1535-8 C_tepidum.fa. The genome-wide protein set of *Chlorobium tepidum* TLS in Fasta format, downloaded from UniProt.
10.1186/s13104-015-1535-8 P_phaeoclathratiforme.fa. The genome-wide protein set of *Pelodictyon phaeoclathratiforme* BU-1, downloaded from UniProt.
10.1186/s13104-015-1535-8 ensembl_cct.fa. The Fasta file containing the genome-wide protein set of *Chlorobium tepidum* TLS from Ensembl Genomes.
10.1186/s13104-015-1535-8 ensembl_ppb.fa. The Fasta file containing the genome-wide protein set of *Pelodictyon phaeoclathratiforme* BU-1 from Ensembl Genomes.
10.1186/s13104-015-1535-8 ensembl_groups.txt. Predicted protein families (groups) output by OrthoMCL for *C. tepidum* TLS and *P. phaeoclathratiforme* BU-1 Ensembl Genomes protein sets.
10.1186/s13104-015-1535-8 ensembl_ unique_cct.pl. The Perl script used to identify the protein families unique to *C. tepidum* from Ensembl Genomes.
10.1186/s13104-015-1535-8 ensembl_uniqe_ppb.pl. The Perl script used to identify the protein families unique to *P. phaeoclathratiforme* from Ensembl Genomes.
10.1186/s13104-015-1535-8 ensembl_1_copy_both_spp.pl. The Perl script used to identify the protein families with one copy in both species from Ensembl Genomes.
10.1186/s13104-015-1535-8 ensembl_multi_cp_both_spp.pl. The Perl script used to identify the protein families with more than one copy for both species from Ensembl Genomes.
10.1186/s13104-015-1535-8 ensembl_1_cp_cct_x_cp_ppb.pl. The Perl script used to identify the protein families containing 1 copy in *C. tepidum* and multiple copies in *P. phaeoclathratiforme* from Ensembl Genomes.
10.1186/s13104-015-1535-8 ensembl_1_cp_ppb_x_cp_cct.pl. The Perl script used to identify the protein families containing 1 copy in *P. phaeoclathratiforme* and multiple copies in *C. tepidum* from Ensembl Genomes.
10.1186/s13104-015-1535-8 unique_cct.pl. Perl script used to identify the protein families unique to *C. tepidum* from UniProt.
10.1186/s13104-015-1535-8 uniqe_ppb.pl. Perl script used to identify the protein families unique to *P. phaeoclathratiforme* from UniProt.
10.1186/s13104-015-1535-8 1_copy_both_spp.pl. Perl script used to identify the protein families with one copy in both species from UniProt.
10.1186/s13104-015-1535-8 multi_cp_both_spp.pl. Perl script used to identify the protein families with more than one copy for both species from UniProt.
10.1186/s13104-015-1535-8 1_cp_cct_x_cp_ppb.pl. Perl script used to identify the protein families containing one copy in *C. tepidum* and multiple copies in *P. phaeoclathratiforme* from UniProt.
10.1186/s13104-015-1535-8 1_cp_ppb_x_cp_cct.pl. Perl script used to identify the protein families containing one copy in *P. phaeoclathratiforme* and multiple copies in *C. tepidum* from UniProt.
10.1186/s13104-015-1535-8 Table_S1.docx. Table S1: The main protein counts of sequence similarity-based protein families (based on UniProt protein sets) between *Chlorobium tepidum* TLS and *Pelodictyon phaeoclathratiforme* BU-1 that do not contain any proteins with functional annotation.

## Abbreviations

*C. tepidum*: *Chlorobium tepidum*
NCBI: National Center for Biotechnology Information
*P. phaeoclathratiforme*: *Pelodictyon phaeoclathratiforme*
rRNA: ribosomal ribonucleic acid
