# Supplementary material for: A comparison of the protein-coding genomes of two green sulphur bacteria, Chlorobium tepidum TLS and Pelodictyon phaeoclathratiforme BU-1
Source: BMC Res Notes. 2015 Oct 14;8:565. doi: 10.1186/s13104-015-1535-8 (PMC4606965; doi:10.1186/s13104-015-1535-8)
Supplement: Supplementary file 19 — 10.1186/s13104-015-1535-8 Table_S1.docx. Table S1: The main protein counts of sequence similarity-based protein families (based on UniProt protein sets) between Chlorobium tepidum TLS and Pelodictyon phaeoclathratiforme BU-1 that do not contain any proteins with functional annotation. [file 13104_2015_1535_MOESM19_ESM.docx]

Table S1: The main protein counts of sequence similarity-based protein families (based on UniProt protein sets) between *Chlorobium tepidum* TLS (‘cct’) and *Pelodictyon phaeoclathratiforme* BU-1 (‘ppb’) that do not contain any proteins with functional annotation.

| Group* | Total Proteins ⱡ | | cct Proteins | ppb Proteins |
| --- | --- | --- | --- | --- |
| 2 | 14 | | 0 | 14 |
| 4 | 8 | | 0 | 8 |
| 5 | 8 | | 0 | 8 |
| 6 | 7 | | 0 | 7 |
| 9 | 7 | | 0 | 7 |
| 11 | 7 | | 0 | 7 |
| 14 | | 6 | 0 | 6 |
| 15 | | 6 | 0 | 6 |
| 16 | | 6 | 0 | 6 |
| 17 | | 6 | 0 | 6 |
| 33 | 4 | | 0 | 4 |
| 36 | 4 | | 0 | 4 |
| 39 | 4 | | 0 | 4 |
| 42 | 4 | | 4 | 0 |
| 29 | 4 | | 1 | 3 |
| 30 | 4 | | 1 | 3 |
| *The group number has been arbitrarily assigned by the OrthoMCL program. Groups are provided in an Additional File.  ⱡ Only family groups with 4 or more orthologous proteins were included. | | | | |
